# C-752-02. Modified Baux Scores and Mortality at a Large Burn Center: A Retrospective Five-Year Institutional Analysis

**DOI:** 10.1093/jbcr/irag033.015

**Published:** 2026-04-06

**Authors:** Mark Shearer, Patrice Sarrazin, Austin Dobson, Bounthavy F Homsombath, Martin Hardy, Zaheed Hassan, Shawn P Fagan, Tayseer Chowdhry, Rajiv S Sood

**Affiliations:** Philadelphia College of Osteopathic Medicine, Moultrie, Georgia; Philadelphia College of Osteopathic Medicine, Moultrie, Georgia; Philadelphia College of Osteopathic Medicine, Moultrie, Georgia; Joseph M. Still Burn Centers, Inc., Augusta, Georgia; Joseph M. Still Burn Centers, Inc., Augusta, Georgia; Joseph M. Still Burn Centers, Inc., Augusta, Georgia; Joseph M. Still Burn Centers, Inc., Augusta, Georgia; Burn and Reconstructive Centers of America, Augusta, Georgia; Burn and Reconstructive Centers of America, Augusta, Georgia

## Abstract

**Introduction:**

The modified Baux score (rBaux) is a useful tool to predict mortality in burn patients due to its easy calculability, accuracy, and potential for clinical utility. This study aims to retrospectively analyze the actual mortality rates at rBaux >100 at a large burn center, with the goal of providing clinicians with a more accurate model for predicting mortality in high-risk burn patients within the institution and facilitating comparison with outcomes reported by other institutions.

**Methods:**

Data from the past 5 years was extracted for 119 patients with Baux scores >100. Patients were stratified into 10-point rBaux intervals (e.g., 100–109, 110–119, etc.), and the mortality rate for each interval was calculated using descriptive statistical methods. Linear regression was used to predict mortality rate at each interval. The burn center’s mortality rate at each rBaux interval was then stratified into two groups, Baux scores without inhalation injury and with inhalation injury, and compared to the data from the National Burn Repository’s 2016 report.

**Results:**

At the burn center, observed mortality rates for rBaux scores >100 ranged from 15.4% (100-109) to 100% (>170). A linear regression model (*p*<.05) predicted increasing mortality with rBaux score, estimating 50% mortality at rBaux 129.

Compared to National Burn Repository (NBR) data, the burn center’s patients with inhalation injury had generally lower mortality at comparable rBaux ranges (ex. 33.3% vs 88.7% at rBaux 120-129.9, and 77.8% vs 96.8% at >140). For patients without inhalation injury, the mortality rates were also lower than the NBR at most rBaux scores >100.

**Conclusions:**

A linear regression model based on past performance was created with an equation of Mortality Rate = 0.0084(rBaux Score)-0.5862 for clinicians to possibly predict mortality rate within the burn center with higher accuracy. The mortality rates for high rBaux score burn patients at the burn center were lower in all score ranges reported by the National Burn Repository except in patients with inhalation injury with rBaux scores of 100-109.9 and 110-119.9, likely due to sample sizes of 0 or 1. Mortality was significantly lower (*p*<.05) at the burn center than national benchmarks in Baux scores 100-109.9, 130-139.9, >140 and rBaux scores 120-129.9, and > 140. These findings may reflect institution-specific strengths such as quick access to ten operating rooms for burns, close integration with critical care and pulmonology services, and a broad array of advanced treatment options for rapid wound closure.

**Applicability of Research to Practice:**

An institutionalized rBaux score has the potential to guide individualized patient care plans, to evaluate the effectiveness of treatment protocols and benchmark institutional performance, to appropriately allocate resources and prioritize interventions, to help optimize management strategies, and finally to support broader clinical decision-making and quality of care.

**Funding for the study:**

N/A.

